# Understanding the relationship between social support and mental health of humanitarian migrants resettled in Australia

**DOI:** 10.1186/s12889-022-14082-z

**Published:** 2022-09-13

**Authors:** Hemavarni Doma, Thach Tran, Pilar Rioseco, Jane Fisher

**Affiliations:** 1 Global and Women’s Health, School of Public Health and Preventive Medicine, Monash University, Melbourne, Victoria Australia; 2 Australian Institute of Family Studies, Melbourne, Victoria Australia

**Keywords:** Humanitarian migrants, Social support, Mental health, Resettlement, Refugees, Asylum seekers

## Abstract

**Background:**

Forced migration can lead to loss of social support and increased vulnerability to psychological distress of displaced individuals. The aims were to ascertain the associations of sociodemographic characteristics and social support received by resettled adult humanitarian migrants in Australia; determine the relationship between social support and mental health at different intervals following humanitarian migration; and examine the modification effects of gender, age and migration pathway on that relationship.

**Methods:**

A secondary analysis was conducted of data generated in Waves One (three to six months after resettlement), Three (three years after resettlement) and Five (five years after resettlement) of the Building a New Life in Australia prospective cohort study. The association between sociodemographic characteristics and mental health were examined at each timepoint using a multivariate regression model. Exploratory factor analysis was used to develop a two-factor social support scale (emotional/instrumental and informational support) from a larger set of items collected in the BNLA. Psychological distress was measured by the Kessler-6 scale. Path analysis was used to analyse the relationships between social support and psychological distress among the three time points considering socio-demographic characteristics simultaneously.

**Results:**

A total of 2264 participants were included in the analyses. Age, gender, birth region, migration pathway, education level and English proficiency were significantly associated with both social support types. Main source of income was only significantly associated with informational support. Remoteness area was only significantly associated with emotional/instrumental support. As emotional/instrumental support increased by one standard deviation (SD) at Wave One, psychological distress at Wave Three decreased by 0.34 score [95% CI (− 0.61; − 0.08)]. As informational support at Wave Three increased by one SD, psychological distress at Wave Five decreased by 0.35 score [95% CI (− 0.69; − 0.01)]. The relationships between social support and psychological distress varied between genders, age groups and migration pathways.

**Conclusion:**

Findings demonstrate the importance of emotional/instrumental support and informational support for the medium and long-term mental health of humanitarian migrants. This study also highlights the important of extending current social support provisions and tailoring programs to enhance support received by humanitarian migrant subgroups years after resettlement to improve mental health.

**Supplementary Information:**

The online version contains supplementary material available at 10.1186/s12889-022-14082-z.

## Background

According to the United Nations High Commissioner for Refugees (UNHCR), more than 80 million people are currently displaced globally [1]. Among them, more than 26 million are refugees and more than 4 million are asylum seekers [1]. Over the past decade, Australia has resettled more than 110,000 refugees, the third-highest number globally behind two other high-income countries, Canada and the United States [2]. Pre- and post-migration experiences can increase the vulnerability of refugees to mental health problems and psychological distress [3, 4]. Yet, whilst the role that trauma has on the mental health of humanitarian migrants has been well-documented [5, 6], the detrimental impact of loss of social connections on mental health after resettlement [3, 7] in high-income countries like Australia is less well described.

Social relationships, or networks, provide social support [8]. Whilst there are several definitions, social support is commonly described as the functional aspect of relationships where resources, assistance and aid are exchanged or provided to an individual through family, friends, community groups, and government services [9]. Forced displacement leads to disruption of social connections for humanitarian migrants and involves rebuilding social networks in the host country [7, 10]. Among Latin American and African refugees in Canada, 80% of refugees experience continued separation from their family members for an average of 3.5 years post-migration [11]. Such disruption can lead to a loss of social support.

The context and structures that influence the provision of social support are essential when discussing social support that is received by an individual [9, 12]. For example, in a group of Chinese and Somali refugees and immigrants in Canada, a country with broadly similar humanitarian settlement services as Australia [13], Stewart [14] found that lack of financial resources (e.g., monetary savings from their country of origin) and language proficiency impeded social support provisions including access to education training in the host country. Having a small or less-established ethnic group in the host country was also a barrier to access social services and support [14]. Importantly, Simich [15] found that recreating social ties and social support, especially with refugees from their ethnic group, was crucial to the emotional wellbeing of refugees.

Social support has become widely considered an essential protective factor for mental health [16, 17]. In a systematic review of 36 studies, social support was consistently associated with protecting adults from depression [17]. Social support has also been shown to be a protective factor of mental health in humanitarian migrant populations [18] including in Syrian refugees where ongoing separation from family, social networks and sources of social support was associated with increased psychological distress [19]. Further, symptoms of depression decreased in refugee groups as sources of support from friends and family increased [16, 20]. Conversely, refugees with weaker social networks and support reported more severe mental health problems [18]. In Australia, in a group of 63 Sudanese refugees in Southeast Queensland, stronger support was a significant predictor of better mental health [3]. Therefore, social support post-migration appears to play a role in shaping the experiences of refugees during resettlement and protecting against adverse psychological distress.

Although the influence of social support on mental health in humanitarian migrant communities has been documented, there remains a gap in the evidence about the relationship between specific types of social support, time since resettlement and mental health among humanitarian migrants residing in a high-income country. Hence, among adult humanitarian migrants resettled in Australia, the aim was to: (1) describe the specific types of social support offered to humanitarian migrants; (2) describe the sociodemographic characteristics associated with receiving social support; (3) determine the relationship between types of social support and mental health at different times after resettlement; and (4) understand the effect modification of gender, age and migration pathway on the relationship between social support and mental health.

## Methods

### Setting

Australia is a high-income country, resettling humanitarian migrants long-term, yearly. However, social and health services for humanitarian migrants may not be immediately provided upon arrival [21].

In Australia, the Humanitarian Settlement Program (HSP) provides support services to humanitarian migrants on permanent protection visas [22]. Permanent protection affords humanitarian migrants the right to work, study and permanently resettle in Australia [22]. Overseen by the Department of Home Affairs, the HSP provides support on services including connecting with community groups, access to housing, English proficiency training, and Medicare [22]. These services are provided by organisations such as Settlement Services International (SSI) and Adult Multicultural Education Services (AMES) Australia across 11 locations in Australia [22]. For example, SSI and AMES provide informational support services on finding employment, education and developing English language skills [23, 24]. They provide instrumental support services including translation services, basic household goods packages and on arrival logistical needs such as transportation from the airport and assistance in finding short-term and long-term accommodation [23, 24]. SSI also assists in emotional support provisions by linking refugees with communities [23]. The support provided via the HSP is short term (six to 18 months) with the expectation that humanitarian migrants will eventually transition to services provided within the community and seek support through other programs, including the Settlement Engagement and Transition Support (SETS) program [22]. The SETS is a government-funded program that aims to support the specific needs of humanitarian migrants [25].

### Building a new life in Australia study

The present study is a secondary analysis of data collected from the Building a New Life in Australia (BNLA) study, a large-scale longitudinal cohort study tracing the settlement journey of humanitarian migrants in all Australian states. Data from the BNLA study are available to researchers.

The BNLA study has been commissioned by the Department of Social Services (DSS) and undertaken by the Australian Institute of Family Studies (AIFS). Detailed information about BNLA has been reported elsewhere [26]. Participants of the BNLA consisted of permanent offshore humanitarian migrants, including refugees (Visa Subclass 200), women-at-risk (Visa Subclass 204) and permanent onshore humanitarian migrants on the protection visa (Visa Subclass 866). People granted permanent protection visas between May and December 2013 (three to six months before the recruitment dates) were eligible.

First, AIFS randomly identified and selected eligible primary visa applicants (PAs) aged 18 years or older from 11 sites in Australia across metropolitan and regional areas using the Settlement Database, which provides statistical data on all permanent arrivals to Australia [27]. Recruitment site was selected by AIFS to ensure each site optimally represented the diversity of humanitarian visa subclasses, and rate of humanitarian migrant settlement [26].

AIFS partnered with Colmar Brunton Social Research (CBSR), and Multicultural Marketing and Management (MMM). Both CBSR and MMM collected the data and conducted the fieldwork for the BNLA study. AIFS supplied the contact details of the principal applicants to CBSR who invited each of them to participate in the study [26]. Following initial contact, CBSR interviewers telephoned each potential participating principal applicant to ascertain their interest in participating in the study and schedule an interview [26]. For each principal applicant who agreed to participate, up to two secondary applicants who were on the same visa as the principal applicant, residing in the same household as the principal applicant, and 15 years or older were randomly selected and invited to participate in the BNLA. A total of 2399 people (principal applicants = 1509, secondary applicants = 890) were recruited.

The BNLA comprises five waves of data collected annually from 2013 to 2018. Data from waves two and four were collected via a questionnaire administered through a computer-assisted telephone interview (CATI) with an interviewer and interpreter present if required by the participant [26]. Data from waves One, Three and Five were collected during home visits by CBSR fieldworkers and interviewers [26]. The questionnaire was administered either via computer-assisted self-interviews (CASI), which used a computer tablet with audio and flashlight function to enable participants to listen to the questions or computer-assisted personal interviews (CAPI), which enabled participants to complete the survey with an interviewer present [26]. Participants were given the option to choose their mode of interview. When neither method was feasible, an accredited interpreter was present over the phone or in person alongside an interviewer to pose questions and record answers [26].

The questionnaire was translated into multiple languages (e.g., Arabic, Persian, Dari) and designed based on the work of the BNLA advisory group comprising experts in different areas such as survey methodology, longitudinal studies, and refugee and migrant studies.

### Participants

This secondary analysis included all primary applicants and secondary applicants aged 18 years or older who provided data for the BNLA. 15- to 17-year-old participants were excluded from this study because the mental health of adolescents and adolescent social support services may differ from adults.

### Data sources

This secondary analysis used data collected in Waves One (baseline, three to six months after resettlement), Three (three years after resettlement) and Five (five years after resettlement).

#### Social support

A seminal work by House [9] categorises social support into four main types. Emotional support is defined as expressions of care, comfort and empathy in social interactions; instrumental support as tangible, task-oriented, and material assistance; informational support as the provision of suggestions, advice and new information; and appraisal support as communicating information relevant to self-evaluation such as constructive feedback [9, 12]. While social support can be conceptually differentiated into four types, some social ties may provide one or more types of support [8].

We created a 10-item scale to measure social support provided to humanitarian migrants using questions in the BNLA questionnaires (Supplementary file 1). Items across the BNLA questionnaires were selected to be included in the scale according to the theory of social support by House [9] and work by Berkman and Glass [28] and Barrera [29]. Exploratory factor analysis was conducted on the selected items. Two subscales (factors) were identified which corresponded to three social support types: emotional, instrumental and informational support. Emotional and instrumental support were measured with the first factor, and informational support with the second factor. Appraisal support was not measured as it was not identified as a factor from the selected items of the BNLA.

The first subscale, emotional/instrumental support, consists of three items assessing the support and comfort provided by a community to assist with resettlement. The scores were summed and standardised (mean = 0 and SD = 1) to create a total emotional/instrumental support score where a high score indicates higher emotional/instrumental support (Supplementary file 2). The internal consistency of the scale was tested using Cronbach’s alpha coefficient where a coefficient > 0.8 indicates high internal reliability. For the emotional/instrumental support subscale, the internal consistency was α = 0.83 at Wave One and α = 0.86 at Wave Three.

The second subscale, informational support, consists of seven items assessing whether information, suggestions and advice on services essential to integrate and function in society have been received. The scores were summed and standardised to create a total informational support score where a high score indicated higher informational support (Supplementary file 2). For the informational support subscale, the internal consistency was α = 0.91 at Wave One and α = 0.92 at Wave Three.

#### Psychological distress

Psychological distress symptoms were assessed using the Kessler-6 scale (K6) that included six items describing depression and anxiety symptoms [30, 31]. The items are scored on a five-point scale: 1 (none of the time), 2 (a little of the time), 3 (some of the time), 4 (most of the time), and 5 (all of the time). The scores were summed to create a total scale score, with a higher score indicating more symptoms of psychological distress. In this study, the internal consistency for the K6 was α =0.89 at Wave One, α =0.90 at Wave Three and α = 0.92 at Wave Five. The scale has also been translated and validated across different languages including in Arabic where the K6 demonstrated high internal consistency (Cronbach’s α =0.81) and high convergent validity with two other scales: the Generalised Anxiety Disorder (GAD-7) and Somatic Symptoms Scale (SSS-8) [32].

#### Socio-demographic characteristics

At baseline, socio-demographic characteristics were collected using study-specific questions on age, gender, marital status, country of birth, remoteness area, education level, and main sources of income.

English proficiency in the BNLA was determined by four items: how well do you (1) understand spoken English, (2) speak English, (3) read English, (4) write English. The items were scored on a four-point scale: 1 (very well), 2 (well), 3 (not well), and 4 (not at all). The items were reverse-scored and recoded into 0 (not at all), 1 (not well), 2 (well), and 3 (very well) and summed to create a total score where a high score indicates a higher proficiency. The internal consistency for this scale is α =0.96 in this study.

Migration pathways were assessed as to whether humanitarian migrants arrived in Australia via the onshore or offshore pathway. The offshore pathway is for those granted permanent protection visas (i.e., Visa Subclasses 200, 201, 202, 203 and 204) before arriving in Australia and would be termed refugees [33]. The onshore pathway is for those granted a permanent protection visa (i.e., Visa Subclass 866) after arrival in Australia and would be termed asylum seekers [33].

### Statistical analysis

Analyses were conducted in three stages. In stage one, the associations between the sociodemographic characteristics and each social support type at every time point were examined using a multivariate regression model, controlling for sociodemographic characteristics.

**Fig. 1:**
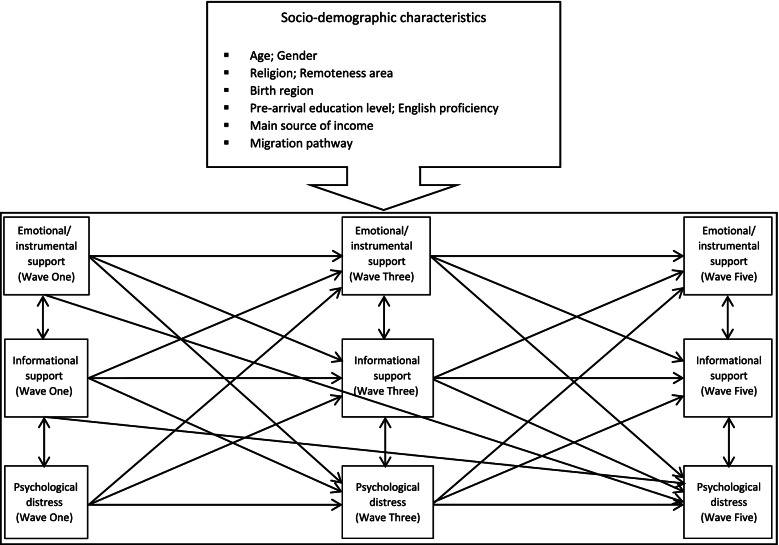
Conceptual framework of the correlation between emotional/instrumental support, informational support and psychological distress

In stage two, a path model was used to analyse the relationship between social support and psychological distress. We developed a conceptual model (Fig. 1) adapted from the model proposed by Heaney and Israel [8] and Watkins et al. [34], and draws upon the social support framework developed by House [9]. This model composes of the directional pathways and correlations between emotional/instrumental support subscale, informational support subscale, psychological distress symptoms, and baseline socio-demographic characteristics at each time point (Waves One, Three, Five). All of the directional pathways and correlations in Fig. 1 were estimated simultaneously. All of the path coefficients were interpreted as linear regression coefficients as all endogenous variables, which were caused by one or more variables in the model, were in continuous scales. The fit of the path model was evaluated using the following criteria: Root Mean Square Error of Approximation (RMSEA) < 0.05, Comparative Fit Index (CFI) ≥ 0.90, and Tucker-Lewis Index (TLI) ≥ 0.90, which indicate a good fit [35].

In stage three, the path model developed in stage two was re-run for subgroups by gender (male and female), age groups (18 to 29 years old, 30 to 44 years old and 45 to 75 years old), and migration pathways [offshore pathway (refugees) and onshore pathway (asylum seekers)] to determine if the effect of social support on psychological distress was modified by gender, age and migration pathway.

The estimation method used for the path models was the maximum likelihood for missing values (MLMV) which accounted for missing data by adjusting the likelihood function to capture information on variables that are observed by cases [36]. All analyses were conducted using Stata Version 16.

### Ethics

Ethics approval for the BNLA study was obtained from the Australian Institute of Family Studies Human Research Ethics Committee (13/03). The Monash University Research Ethics Committee granted ethics exemption to use the data.

## Results

### Participant characteristics

Among 2399 participants of the BNLA, 2264 (1509 PAs and 738 SAs) were eligible for this study. We excluded 135 people who were < 18 years old. All 2264 participants provided data three to six months after resettlement (Wave One). Among those, 1779 (1155 PAs and 624 SAs) were followed-up three years after resettlement (Wave Three) and 1765 (1144 PAs and 621 SAs) five years after resettlement (Wave Five). Hence, the loss to follow-up rate from three to six months to five years after resettlement is 22.1%, with 77.9% of respondents from Wave One retained.

The mean age of participants included in this study was 36.6 years (Table 1). More than half of the participants were men. Humanitarian migrants arrived in Australia from five regions, most were from the Middle East. Approximately 60% of participants were married. Most participants arrived via Australia’s offshore settlement pathway, received government payments and lived in major cities.

**Table 1:** Demographic characteristics at baseline (*n* = 2264)

	Statistics
**Age, mean (SD)**	36.6 (13.4)
**Gender, n (%)**	
Men	1249 (55.2%)
Women	1015 (44.8%)
**Marital status, n (%)**	
No	888 (39.2%)
Yes	1376 (60.8%)
**Birth region, n (%)**	
Africa	149 (6.6%)
Middle East	1197 (52.9%)
South East Asia	134 (5.9%)
Southern Asia	205 (9.1%)
Central Asia	574 (25.4%)
**English Proficiency score, mean (SD)**	4.43 (3.26)
**Education level prior to arrival, n (%)**	
6 or less years of schooling	815 (36%)
7 to 11 years of schooling	606 (26.8%)
12 or more years of schooling	821 (36.3%)
**Remoteness area, n (%)**	
Major cities of Australia	2052 (90.6%)
Regional Australia	212 (9.4%)
**Migration pathway, n (%)**	
Onshore	377 (16.7%)
Offshore	1887 (83.4%)
**Main source of income, n (%)**	
Respondent’s own salary or spouse’s/partner’s/parent’s salary or other	229 (10.3%)
Government payments	2002 (89.7%)
**Mode of interview, n (%)**	
Computer-assisted self-interview	1582 (69.9%)
Computer-assisted personal interview with interviewer	633 (28%)
Computer-assisted personal interview with interpreter	49 (2.1%)

Younger humanitarian migrants were more likely to have received both types of social support than those who were older (Tables 2 and 3). Compared to men, women had received more emotional/instrumental support and less informational support at each Wave. Humanitarian migrants who were married had been given more emotional/instrumental support three years after resettlement than those who were not married (Table 2).

**Table 2:** Association between demographic characteristics (baseline) and emotional/instrumental support at each time point

	Wave One			Wave Three			Wave Five		
	Coef.^**a**^	95% CI	***p***-value^†^	Coef.^**a**^	95% CI	***p***-value^†^	Coef.^**a**^	95% CI	***p***-value^†^
Age (in 10 years)	−0.002	(− 0.010 to 0.061)	0.667	− 0.215	(− 0.302 to − 0.128)	0.000	− 0.123	(− 0.216 to − 0.030)	0.009
Gender									
Men	Ref								
Women	0.257	(0.063 to 0.451)	0.009	0.235	(0.019 to 0.452)	0.033	0.300	(0.069 to 0.530)	0.011
Marital status									
No	Ref								
Yes	0.112	(−0.089 to 0.314)	0.273	0.309	(0.081 to 0.538)	0.008	0.090	(−0.154 to 0.334)	0.471
Birth region									
Africa	Ref								
Middle East	−0.106	(− 0.497 to 0.285)	0.596	0.182	(−0.319 to 0.683)	0.476	−0.653	(−1.203 to − 0.102)	0.020
South-East Asia	−0.406	(− 0.943 to 0.132)	0.139	− 0.182	(− 0.839 to 0.474)	0.586	− 0.930	(− 1.671 to − 0.189)	0.014
Southern Asia	0.297	(− 0.193 to 0.788)	0.235	0.017	(−0.575 to 0.610)	0.954	0.127	(−0.537 to 0.792)	0.707
Central Asia	−0.990	(−1.413 to − 0.566)	0.000	− 0.895	(− 1.427 to − 0.363)	0.001	−0.898	(−1.485 to − 0.311)	0.003
English proficiency (score)	0.132	(0.095 to 0.169)	0.000	0.0676	(0.027 to 0.109)	0.001	0.091	(0.047 to 0.136)	0.000
Education (pre-arrival)									
6 or fewer years of schooling	Ref								
7 to 11 years of schooling	−0.288	(−0.548 to −0.027)	0.031	0.061	(−0.229 to 0.352)	0.679	−0.057	(−0.363 to 0.245)	0.705
12 or more years of schooling	−0.508	(−0.790 to − 0.225)	0.000	−0.076	(− 0.389 to 0.237)	0.634	− 0.404	(− 0.740 to − 0.069)	0.018
Remoteness area									
Major cities	Ref								
Regional Australia	0.547	(0.203 to 0.890)	0.002	0.208	(−0.167 to 0.584)	0.277	−0.181	(−0.580 to 0.218)	0.374
Migration pathway									
Onshore	Ref								
Offshore	0.153	(−0.121 to 0.427)	0.274	0.904	(0.575 to 1.234)	0.000	0.549	(0.185 to 0.914)	0.003
Main source of income									
Own or spouse/parent’s salary, savings	Ref								
Government payments	0.020	(−0.313 to 0.353)	0.906	0.135	(−0.246 to 0.515)	0.488	0.033	(−0.257 to 0.323)	0.824

^†^Statistical significance set at *p* < 0.05

^a^Multiple regression coefficient

**Table 3:** Association between demographic characteristics (baseline) and informational support at each time point

	Wave One			Wave Three			Wave Five		
	Coef.^**a**^	95% CI	***p***-value^†^	Coef.^**a**^	95% CI	***p***-value^†^	Coef.^**a**^	95% CI	***p***-value^†^
Age (in 10 years)	−0.647	(− 0.828 to − 0.465)	0.000	−0.979	(−1.183 to − 0.774)	0.000	−1.765	(−1.991 to −1.540)	0.000
Gender									
Men	Ref								
Women	−1.674	(−2.121 to − 1.226)	0.000	−2.459	(− 2.970 to − 1.948)	0.000	− 2.639	(−3.198 to − 2.081)	0.000
Marital status									
No	Ref								
Yes	0.312	(−0.155 to 0.778)	0.190	− 0.087	(− 0.624 to 0.451)	0.752	0.434	(−0.158 to 1.026)	0.151
Birth region									
Africa	Ref								
Middle East	−2.169	(−3.009 to −1.247)	0.000	−1.117	(−2.313 to 0.078)	0.067	−2.088	(−3.440 to −0.737)	0.002
South-East Asia	−2.221	(−3.471 to −0.970)	0.001	−2.280	(−3.824 to −0.736)	0.004	−5.764	(−7.556 to − 3.973)	0.000
Southern Asia	−1.051	(−2.188 to 0.087)	0.070	−2.942	(−4.351 to −1.533)	0.000	− 2.710	(−4.331 to − 1.090)	0.001
Central Asia	−1.078	(− 2.077 to −0.078)	0.035	−0.834	(− 2.101 to 0.433)	0.197	−2.398	(−3.832 to − 0.964)	0.001
English proficiency (score)	0.628	(0.542 to 0.713)	0.000	0.600	(0.503 to 0.697)	0.000	0.441	(0.334 to 0.549)	0.000
Education (pre-arrival)									
6 or fewer years of schooling	Ref								
7 to 11 years of schooling	0.145	(−0.458 to 0.749)	0.637	− 0.037	(− 0.723 to 0.648)	0.915	0.393	(− 0.342 to 1.129)	0.294
12 or more years of schooling	0.825	(0.172 to 1.478)	0.013	1.156	(0.419 to 1.892)	0.002	1.723	(0.914 to 2.533)	0.000
Remoteness area									
Major cities	Ref								
Regional Australia	−0.497	(−1.281 to 0.287)	0.214	0.868	(−0.025 to 1.762)	0.057	0.672	(−0.294 to 1.639)	0.173
Migration pathway									
Onshore	Ref								
Offshore	−1.036	(−1.672 to −0.400)	0.001	−1.111	(− 1.893 to − 0.330)	0.005	− 0.633	(−1.505 to 0.239)	0.154
Main source of income									
Own or spouse/parent’s salary, savings	Ref								
Government payments	−1.549	(−2.326 to −0.773)	0.000	−0.782	(−1.681 to 0.118)	0.088	−0.752	(−1.451 to − 0.054)	0.035

^†^Statistical significance set at *p* < 0.05

^a^Multiple regression coefficient

The association between birth region and emotional/instrumental support was most significant five years after resettlement, where humanitarian migrants born in the Middle East, South-East Asia and Central Asia received less of this support type than those born in Africa (Table 2). Those born in Central Asia were provided with less emotional/instrumental support than those born in Africa across all Waves. Whilst those born in the Middle East and South-East Asia appeared to receive less emotional/instrumental support than those born in Africa, the difference was not statistically significant three to six months and three years after resettlement. The association became significant five years after resettlement.

Five years after resettlement, all humanitarian migrants born in regions other than Africa received less informational support compared to those born in Africa. Those born in South-East Asia received less informational support than those born in Africa across all Waves (Table 3).

Humanitarian migrants more proficient in English receive more emotional/instrumental and informational support at each Wave. For educational level pre-arrival to Australia, compared to those with six or fewer years of schooling, humanitarian migrants with 12 or more years of schooling received less emotional/instrumental support than those with six or fewer years of schooling three to six months and five years after resettlement but received more informational support at all time points after resettlement (Tables 2 and 3).

Three to six months after resettlement, humanitarian migrants residing in regional Australia received more emotional/instrumental support compared to those residing in major cities (Table 2). Humanitarian migrants with primary income of government payments received less informational support three to six months and five years after resettlement than those reliant on their own salary or from another source (Table 3).

Humanitarian migrants who came to Australia via the offshore pathway (refugees) received more emotional/instrumental support compared to those who came to Australia via the onshore pathway (asylum seekers) three and five years after resettlement (Table 2). Humanitarian migrants arriving as refugees received less informational support than those arriving as asylum seekers three to six months and three years after resettlement (Table 3).

### Relationship between social support and mental health

The path model (Table 4) of the relationships between social support types and mental health at different time points had a good fit (CFI = 0.98, TLI = 0.90, RMSEA = 0.03).

**Table 4:** Relationship between social support and psychological distress

Pathway	Path coef.^a^	95% CI	*p*-value^†^
Emotional/instrumental support Wave One → Psychological distress Wave Three	−0.342	(− 0.607 to − 0.077)	0.012
Informational support Wave One → Psychological distress Wave Three	0.000	(−0.308 to 0.308)	0.999
Emotional/instrumental support Wave One → Psychological distress Wave Five	−0.192	(−0.467 to 0.084)	0.173
Informational support Wave One → Psychological distress Wave Five	−0.313	(−0.641 to 0.015)	0.062
Emotional/instrumental support Wave Three → Psychological distress Wave Five	−0.006	(−0.294 to 0.281)	0.965
Informational support Wave Three → Psychological distress Wave Five	−0.347	(−0.689 to − 0.005)	0.047

^†^Statistical significance set at *p* < 0.05

^a^Path coefficients were estimated simultaneously using a path model controlling for all socio-demographic characteristics in Table 1. For full details of this model see Supplementary file 2: Table 1

The emotional/instrumental support received by humanitarian migrants three to six months after resettlement affected psychological distress three years after resettlement. As the emotional/instrumental support increased by one SD three to six months after resettlement, psychological distress (K6 score) three years after resettlement decreased by 0.34 score. The information support received three years after resettlement influenced psychological distress three years after resettlement. As informational support three years after resettlement increased by one SD, the psychological distress score five years after resettlement decreased by 0.35 (Table 4).

### Effect modification of gender, age and migration pathway on the relationship between social support and mental health

The relationships between social support and psychological distress were slightly different between men and women. Informational support influenced the severity of psychological distress score in both groups but at different time points. Psychological distress five years after resettlement decreased as the informational support received by men increased three years after resettlement. In contrast, psychological distress five years after resettlement decreased as the informational support received by women increased three to six months after resettlement (see Supplementary file 2: Table 2).

The relationships between social support and psychological distress varied among the three age groups. The severity of psychological distress three years after resettlement decreased as the emotional/instrumental support received by those aged between 18 and 29 increased three to six months after resettlement. The severity of psychological distress symptoms five years after resettlement decreased as the informational support received by humanitarian migrants aged 45 to 75 years old increased three to six months after resettlement. No significant effects of social support on psychological distress were found among people aged between 30 to 44 (see Supplementary file 2: Table 3).

For the relationship between social support types and psychological distress among onshore (asylum seekers) and offshore (refugees) participants, psychological distress decreased five years after resettlement as the informational support received by asylum seekers increased three years after resettlement. Psychological distress decreased three years after resettlement as emotional/instrumental support received by refugees increased three to six months after resettlement. Psychological distress decreased five years after resettlement as the informational support received by refugees increased three to six months after resettlement (see Supplementary file 2: Table 4).

## Discussion

To our knowledge, this is the first longitudinal study of experiences of specific social support types received by humanitarian migrants and their mental health in a high-income host country.

### Medium- and long-term benefits for mental health

Our findings show that increased emotional/instrumental support received in the initial phase of resettlement (three to six months) may be more beneficial for mental health in the medium-term (first three years of resettlement). This observation could reflect the sudden loss in emotional and instrumental support from family and community that humanitarian migrants feel after displacement, which may be more pronounced during the initial years of resettlement [37]. This loss of support may increase psychological distress as experienced by resettled Sudanese refugees in Canada [37]. Our findings also demonstrate that more informational support received three years after resettlement is beneficial for the long-term mental health (five years after resettlement) of humanitarian migrants. Hence, knowledge on how to integrate and thrive in society may enable a greater sense of control over their lives and improve long-term mental health [8].

For humanitarian migrant women, we found more informational support received within three to six months of resettlement improved mental health five years after resettlement. This finding suggests more focused informational support in the initial months of resettlement is beneficial for the long-term mental health of women. As observed in a group of Syrian refugee women, those who received more informational support when they were first resettled were able to problem-solve more efficiently and had greater control over their lives which reduced psychological distress [8, 38].

For men, informational support received three years after resettlement was beneficial for their mental health five years after resettlement. This observation was not made between social support types received in the initial months of resettlement and long-term mental health. Hence, our finding may indicate that informational support is more important for mental health of men years into resettlement after they would have exited formal services such as the HSP.

Among humanitarian migrants aged 18 to 29 years old, emotional/instrumental support received three to six months after resettlement was beneficial for mental health three years after resettlement. In contrast, among humanitarian migrants aged between 45 to 75 years old, informational support received three to six months after resettlement was beneficial for mental health five years after resettlement. Our findings suggest different types of social support provisions may benefit the mental health of younger and older humanitarian migrants [39]. Minicuci et al. [40] found less received financial support to be significantly associated with depression in older men; however, emotional support provisions were not associated with mental health [40]. In Australia, Bartolomei [41] highlights insufficient provisions of support after resettlement, such as knowledge of how systems operate and the rights of refugees, and lack of clear advice on how to access benefits and seek employment affected the mental health of older Sudanese refugees. We found no effect of social support on psychological distress among people aged between 30 to 44. Other forms of support not included in this study, such as financial or childcare support, could have a greater effect on the mental health of humanitarian migrants aged between 30 and 44, given that this age group could most likely be parents and in paid employment. However, further research would be needed to determine whether these forms of support are associated with mental health in this age group.

For humanitarian migrants with refugee status, informational support received three to six months after resettlement was beneficial for their mental health five years after resettlement. For humanitarian migrants who arrived in Australia as asylum seekers, we found informational support received three years after resettlement improved their mental health five years after resettlement. We also found more emotional/instrumental support received three to six months after resettlement improved the mental health of refugees three years after resettlement. Our observations indicate that specific sources of emotional/instrumental and informational support received by refugees in the initial months of resettlement are beneficial for their medium- and long-term mental health. However, asylum seekers may require more informational support provisions years into resettlement to benefit their long-term mental health.

### Subgroups who receive more (or less) specific social support types

Our findings demonstrate that those with lower English proficiency received less of each form of social support at all time points. Older humanitarian migrants received less informational support across all times and less emotional/instrumental support three and five years after resettlement.

Women received more emotional/instrumental support compared to men across all time points, and humanitarian migrants residing in regional Australia than in major cities within the initial months of resettlement. Those with refugee status also received more emotional/instrumental support three and five years after resettlement than those who arrived as asylum seekers.

Humanitarian migrants born in Central Asia received less emotional/instrumental support than humanitarian migrants born in Africa at every time point. Those with 12 or more years of schooling also received less emotional/instrumental support compared to those with six or fewer years of schooling during initial resettlement and after five years of resettlement.

More informational support was provided to men than women and individuals with 12 or more years of education compared to those with six or fewer years of schooling at each time point.

Less informational support was provided to humanitarian migrants who received government payments as their main source of income than those who were reliant on their own or their spouse’s salary or savings when they were initially resettled and at five years after resettlement. Less informational support was also provided to humanitarian migrants who arrived in Australia as refugees compared to those who arrived as asylum seekers in the first five years after resettlement, and those born in South-East Asia than humanitarian migrants born in Africa across all time points.

### Why certain subgroups may receive more (or less) social support

The Australian Government provides mainstream social support services to Australians, which includes health and aged care services, housing, transportation, education, employment and training, and childcare and support [42]. Accessibility to these services is often facilitated by financial benefits delivered predominantly by the Centrelink Master Program, an Australian Government agency providing government payments and services for eligible groups such as students, the unemployed, and people from diverse cultural and linguistic backgrounds [43]. Employment support is facilitated by the Australian Government’s mainstream employment service Jobactive, which seeks to connect job seekers with employers [44]. Furthermore, all Australians have access to Medicare, the universal health care insurance scheme in Australia [45]. While humanitarian migrants have access to specialised settlement services upon arrival, such as the HSP and SETS, they may also be able to access these mainstream services. However, eligibility for these mainstream support services depends on humanitarian migrants’ visa type [46].

Refugees and humanitarian migrants with a valid visa are granted the right to reside temporarily or permanently, work, and study [47]. They may also have access to government benefits such as Centrelink, Jobactive and Medicare [47]. However, those who have entered Australia without a valid visa and are, thus, seeking asylum and awaiting the outcome of their visa application, may not have access to most formal, mainstream social services [48]. Instead, they can access the Status Resolution Support Services (SRSS), a program that offers temporary needs-based support in accessing accommodation, healthcare and education for children, and financial benefits [49, 50].

In addition to mainstream support services, Australians also receive informal sources of support from their neighbours, family, and social networks [51]. In 2021, 10% of Australians provided informal support as an informal carer, a person who assists in carrying out tasks, provides transportation, and in-home supervision [52]. In addition, 24% of Australians also provided informal support through volunteering with organisations such as sports, recreation, education and training [51]. Though these informal sources of support are often less accessible by humanitarian migrants, especially those newly-arrived or with no pre-existing connections such as extended family, existing ethnic community in the host country [53].

From our findings, in Australia, older humanitarian migrants may be provided with less social support because they may find it more difficult to navigate support services such as finding accommodation and accessing financial support [41]. This could be due to a lack of knowledge or understanding of how the Australian system functions compared to younger humanitarian migrants [41]. This support may not be readily provided or available to them [41]. Older Sudanese refugees have reported not having anyone to help them in their neighbourhood after resettlement in Australia [41].

Humanitarian migrants who are more proficient in English may know how to navigate support systems more effectively than those with less proficiency in English. Studies have demonstrated the difficulty experienced by migrants in engaging with services without some fluency in the host language, which limits access to resources [39, 54]. The lack of readily available and affordable interpreters to assist humanitarian migrants in engaging with services may further limit access to services for those with lower English proficiency [55]. The Adult Migrant English Program (AMEP) in Australia provides free English language training to permanent protection visa holders to improve their English skills. However, only a maximum of 510 hours of English tuition were offered to each humanitarian migrant throughout the BNLA study period [56]. This was insufficient for humanitarian migrants to gain a level of proficiency required for success in employment and education [34]. Since April 2021, the AMEP has been reformed to allow unlimited English tuition until migrants reach a level of English needed to succeed in vocational education and training [56].

Humanitarian migrant men were provided with less emotional/instrumental support services and more informational support than women which could indicate a difference in service provision between men and women [8, 57]. The complex nature of service delivery and lack of flexible access to services catering to the specific circumstances of humanitarian migrant women [58] could explain why they received less informational support than men. In Australia, resettled refugee and migrant women may bear greater responsibility than men in maintaining the household and providing childcare, which could impede their access to English language training, and finding and retaining employment [58]. This dynamic can persist years into resettlement and can often result in women finding precarious employment whilst lacking sufficient knowledge on their rights, and how to seek secure employment [58].

In an online survey of service providers, De Maio et al. [58] also demonstrates that humanitarian migrant women face barriers such as low English language skills, location and transport issues, and family responsibilities to a greater extent than Australian-born women when accessing support services. This is reflected in humanitarian migrant women’s lower employment rate than Australian-born women when comparing women with similar education levels and the same number of children [58]. While services are available to migrant women, such as language classes and parenting support, they may not be aware of these services nor confident in accessing them [58]. Men may receive less emotional/instrumental support because services that provide such support may not be readily available and accessible to men as they may be to women. Our finding is consistent with previous studies demonstrating that women offer and receive more emotional support than migrant men [59, 60]. While migrant and refugee women are also more likely to seek help than men [61], further research is needed on why humanitarian migrant men may receive less emotional/instrumental support.

Humanitarian migrants residing in regional areas of Australia received more emotional/instrumental support within the initial months of resettlement which could be explained by the regional resettlement scheme, a government-led effort to revitalise regional areas and provide a welcoming community [62]. Refugees residing in regional areas of Australia have identified friends, family and a welcoming community as reasons to remain in those regions [62].

Differences in provisions of social support for humanitarian migrants may also be present for those with different cultural backgrounds. From 2010 to 2011, many government grants for the SETS program focused on African communities [63]. These grants may have assisted in supporting African communities in Australia and could explain the higher level of support received by African communities compared to other ethnic communities [64]. Furthermore, in Australia, De Maio et al. [58] found that service providers may lack training on the cultural specificity of different humanitarian migrant ethnic groups which is required to deliver appropriate and adequate support. Our finding is also consistent with a study in the United States demonstrating the variation in social support provided from family and friends to immigrant minority groups based on ethnicity [65].

Humanitarian migrants with six or fewer years of education or who are reliant on government payments may find it challenging to engage with support services. These services may not be designed to accommodate a range of education levels or financial situations [41, 55]. For example, humanitarian migrants often need to pay for an interpreter when engaging with services as they are not readily provided [55]. Furthermore, low education levels, unemployment and low income have been associated with less received support [60]. In contrast, humanitarian migrants with six or fewer years of education may be provided with more emotional/instrumental support compared to those with 12 or more years of education which could indicate more focused emotional/instrumental support from providers for those who are less educated. However, further investigation into this observation may be needed to understand why those with fewer years of education received more emotional/instrumental support.

Similarly, further investigation into the difference in social support provisions between refugees and asylum seekers on permanent protection visas is needed. Our findings indicate asylum seekers may not be provided with sufficient emotional/instrumental support within the first three years of resettlement and those with refugee status may not be provided with sufficient informational support three or more years after resettlement. However, literature on the difference in social support service provision between humanitarian migrants who arrive in a host country as refugees or asylum seekers is limited.

### Implications for resettlement support services

Our findings indicate access to support through resettlement services may be needed after 18 months as it could be beneficial for both the medium- and long-term mental health of humanitarian migrants. Our findings also demonstrate the importance of increasing provision of specific social support types for each subgroup at different times after resettlement for both medium- and long-term mental health.

Services delivered in a gender-sensitive manner may assist in providing more emotional/instrumental support for men and informational support for women. For men and humanitarian migrants who arrived in Australia as asylum seekers, more tailored informational support services may be needed three years after resettlement for their long-term mental health. For humanitarian migrants with refugee status and younger humanitarian migrants aged 18 to 29 years old, more emotional/instrumental support services may be needed in the initial months of resettlement for their medium-term mental health. For women, older humanitarian migrants aged between 45 to 75 years old, and humanitarian migrants who arrived in Australia as refugees, more informational support services provided in the initial months of resettlement could benefit their long-term mental health.

More emotional/instrumental and informational support provisions by resettlement services would benefit men and women, respectively. Social support providers also may tailor services to accommodate humanitarian migrants with lower education levels. It may also be important to increase focus towards services provided in a culturally and linguistically appropriate manner for humanitarian migrants with lower English proficiency and of different cultural backgrounds. Furthermore, our findings also indicate that strategies may be needed on how to support older humanitarian migrants in their new community. Support providers may not have the appropriate services tailored towards their specific needs [41].

### Strengths and limitations

The major strengths of this study are the large cohort size and prospective nature which allowed us to look at the change in the relationship of social support and mental health at multiple times, thus, providing more robust evidence for causality. Another strength is the nationally-representative sample population which allows for generalisability of the outcomes to humanitarian migrants with permanent protection visas residing across Australia.

We acknowledge that we could not fully capture the components of social support as this was a secondary analysis of existing data. We also recognise that the rates of attrition were high in this study. As the missing data occurred in all major variables, MLMV was used to handle the missing values, which is the most suitable method to treat missing data for this study. The missing data will not significantly affect the study results because there is no indication that the missing values were not at random. We also do not know how many humanitarian migrants in our sample population were using the services available through the HSP throughout the study period. Finally, we acknowledge that our reasoning behind why certain groups received more or less support is speculative as evidence is currently limited or not available.

## Conclusion

Our study demonstrates the importance of emotional/instrumental and informational support for the medium- and long-term mental health of humanitarian migrants. It also highlights the need to extend social support provisions offered by the HSP beyond the current limit of 18 months. Our study also demonstrates the importance of tailoring support services for subgroup populations of humanitarian migrants, which may benefit their mental health years into resettlement.

## Supplementary Information

**Additional file 1: Supplementary file 1: Table 1.** Building a New Life in Australia social support scale. **Table 2.** Factorial structure of the Building a New Life in Australia social support scale. **Supplementary file 2: Table 1.** Relationship between social support and psychological distress (full path model). **Table 2.** Effect of gender on social support and psychological distress (full path model). **Table 3.** Effect of age group on social support and psychological distress (full path model). **Table 4.** Effect of migration pathway on social support and psychological distress (full path model).

## Data Availability

The data that support the findings of this study are openly available upon request and with permission from the National Centre for Longitudinal Data, Australian Government Department of Social Services at https://dataverse.ada.edu.au/dataverse.xhtml?alias=bnla.
