# Supplementary material for: Understanding the relationship between social support and mental health of humanitarian migrants resettled in Australia
Source: BMC Public Health. 2022 Sep 13;22:1739. doi: 10.1186/s12889-022-14082-z (PMC9472377; doi:10.1186/s12889-022-14082-z)
Supplement: Supplementary file 1 — Additional file 1: Supplementary file 1: Table 1. Building a New Life in Australia social support scale. Table 2. Factorial structure of the Building a New Life in Australia social support scale. Supplementary file 2: Table 1. Relationship between social support and psychological distress (full path model). Table 2. Effect of gender on social support and psychological distress (full path model). Table 3. Effect of age group on social support and psychological distress (full path model). Table 4. Effect of migration pathway on social support and psychological distress (full path model). [file 12889_2022_14082_MOESM1_ESM.docx]

# Supplementary file 1

**Table 1. Building a New Life in Australia social support scale**

| Subscale 1 – emotional/instrumental support | No (0) | | Sometimes (1) | | Yes (2) | |
| --- | --- | --- | --- | --- | --- | --- |
| Given support/comfort from national or ethnic community |  | |  | |  | |
| Given support/comfort from religious community |  | |  | |  | |
| Given support/comfort from other community groups |  | |  | |  | |
| Subscale 2 – informational support | **Wouldn’t know at all (0)** | **Would know a little (1)** | | **Would know fairly well (2)** | | **Would know very well (3)** |
| Know how to look for job |  |  | |  | |  |
| Know how to use public transport |  |  | |  | |  |
| Know how to get help in an emergency |  |  | |  | |  |
| Know how to use bank services |  |  | |  | |  |
| Know how to use government services |  |  | |  | |  |
| Know how to get help from police |  |  | |  | |  |
| Know how to find out about rights |  |  | |  | |  |

**Social support subscale definition**

Emotional/instrumental support was defined as support and comfort provided by a community to assist with resettlement. The comfort aspect referred to the emotional support side, and the support aspect referred to aspects of instrumental support. Informational support was defined as whether they had received the information, suggestions and advice on services essential to function in society.

**Social support scale development**

Firstly, each item in the BNLA Wave one questionnaire was assessed and items were selected to be included in the scale according to the theory of social support by House (1) and work by Berkman and Glass (2) and Barrera (3) and consistency across Waves One, Three and Five. Items that had a high rate of many missing values or were not directly relevant to the social support framework were excluded from the scale.

Secondly, exploratory factor analysis was conducted on the selected items. The factorial structure of the scale was determined using the baseline social support items at Wave One (baseline) (Table 2). The number of factors selected was decided based on the Kaiser criterion (eigenvalue > 1). If more than one factor met the Kaiser criterion, the Scree plot and total percent variance explained were used to reduce the number of factors. Items with a factor loading of < 0.3 were interpreted as not salient and was omitted from the final version of the social support scale. Ultimately, two factors were identified and the oblique (Promax) factor rotation was applied as we were assuming the factors were related to each other and, therefore, should be correlated.

**References**

1. House JS. Work stress and social support: Addison-Wesley Pub. Co.; 1983.

2. Berkman LF, Glass T. Social integration, social networks, social support, and health. Social epidemiology. 2000;1(6):137-73.

3. Barrera Jr M. Social support research in community psychology. Handbook of community psychology. Dordrecht, Netherlands2000. p. 215-45.

**Table 2. Factorial structure of the Building a New Life in Australia social support scale**

| Social support | | | |
| --- | --- | --- | --- |
| Items/subscales | **Factors** | | **Uniqueness** |
|  | **1** | **2** |  |
| Proportion (variance explained by factor) | 74.30% | 32.10% |  |
| Subscale 1 – Emotional/instrumental support | | | |
| Given support/comfort from national or ethnic community | -0.03 | 0.78 | 0.39 |
| Given support/comfort from religious community | -0.02 | 0.82 | 0.32 |
| Given support/comfort from other community groups | 0.05 | 0.68 | 0.53 |
| Subscale 2 – Informational support | | | |
| Know how to look for job | 0.67 | 0.06 | 0.55 |
| Know how to use public transport | 0.77 | -0.07 | 0.42 |
| Know how to get help in an emergency | 0.83 | -0.07 | 0.32 |
| Know how to use bank services | 0.79 | -0.02 | 0.37 |
| Know how to use government services | 0.8 | 0.09 | 0.34 |
| Know how to get help from police | 0.8 | -0.05 | 0.37 |
| Know how to find out about rights | 0.76 | 0.07 | 0.40 |

# Supplementary file 2

**Table 1. Relationship between social support and psychological distress (full path model)**

| Pathway | Path coef. | 95% CI | *p*-value^ꝉ^ |
| --- | --- | --- | --- |
| Emotional/instrumental support Wave One → Psychological distress Wave Three | -0.342 | (-0.607 to -0.077) | 0.012 |
| Informational support Wave One → Psychological distress Wave Three | 0.00 | (-0.308 to 0.308) | 0.999 |
| Psychological distress Wave One → Psychological distress Wave Three | 0.347 | (0.303 to 0.391) | 0.001 |
| Age → Psychological distress Wave Three | 0.026 | (0.005 to 0.047) | 0.013 |
| Gender → Psychological distress Wave Three |  | |  |
| Men | Ref | |  |
| Women | 0.848 | (0.328 to 1.369) | 0.001 |
| Marital status → Psychological distress Wave Three |  | |  |
| No | Ref | |  |
| Yes | -0.255 | (-0.792 to 0.282) | 0.352 |
| Birth region → Psychological distress Wave Three |  | |  |
| Africa | Ref | |  |
| Middle East | 2.910 | (1.704 to 4.116) | 0.000 |
| South-East Asia | -1.396 | (-2.941 to 0.149) | 0.077 |
| Southern Asia | -1.135 | (-2.529 to 0.259) | 0.110 |
| Central Asia | 0.556 | (-0.726 to 1.839) | 0.395 |
| English proficiency → Psychological distress Wave Three | -0.084 | (-0.187 to 0.019) | 0.108 |
| Education → Psychological distress Wave Three |  | |  |
| 6 or fewer years of schooling | Ref | |  |
| 7 to 11 years of schooling | 0.234 | (-0.448 to 0.916) | 0.501 |
| 12 or more years of schooling | 0.154 | (-0.590 to 0.899) | 0.685 |
| Remoteness area → Psychological distress Wave Three |  | |  |
| Major cities | Ref | |  |
| Regional Australia | 2.086 | (1.198 to 2.975) | 0.000 |
| Migration pathway → Psychological distress Wave Three |  | |  |
| Onshore | Ref | |  |
| Offshore | 0.216 | (-0.568 to 1.000) | 0.589 |
| Main source of income → Psychological distress Wave Three |  | |  |
| Own or spouse/parent's salary, savings | Ref | |  |
| Government payments | -0.350 | (-1.249 to 0.549) | 0.450 |
| Mode of interview → Psychological distress Wave Three |  | |  |
| Computer-assisted self-interview | Ref | |  |
| Computer-assisted personal interview with interviewer | 0.379 | (-0.181 to 0.938) | 0.185 |
| Computer-assisted personal interview with interpreter | -0.528 | (-2.362 to 1.306) | 0.572 |
| Emotional/instrumental support Wave One → Psychological distress Wave Five | -0.192 | (-0.467 to 0.084) | 0.173 |
| Informational support Wave One → Psychological distress Wave Five | -0.313 | (-0.641 to 0.015) | 0.062 |
| Emotional/instrumental support Wave Three → Psychological distress Wave Five | -0.006 | (-0.294 to 0.281) | 0.965 |
| Informational support Wave Three → Psychological distress Wave Five | -0.347 | (-0.689 to -0.005) | 0.047 |
| Psychological distress Wave One → Psychological distress Wave Five | 0.150 | (0.102 to 0.199) | 0.000 |
| Psychological distress Wave Three → Psychological distress Wave Five | 0.296 | (0.245 to 0.347) | 0.000 |
| Age → Psychological distress Wave Five | 0.026 | (0.003 to 0.048) | 0.024 |
| Gender → Psychological distress Wave Five |  | |  |
| Men | Ref | |  |
| Women | 0.057 | (-0.493 to 0.607) | 0.838 |
| Marital status → Psychological distress Wave Five |  | |  |
| No | Ref | |  |
| Yes | -0.651 | (-1.209 to -0.092) | 0.022 |
| Birth region → Psychological distress Wave Five |  | |  |
| Africa | Ref | |  |
| Middle East | 1.520 | (0.324 to 2.717) | 0.013 |
| South-East Asia | -1.120 | (-2.723 to 0.483) | 0.171 |
| Southern Asia | -0.175 | (-1.641 to 1.291) | 0.815 |
| Central Asia | 0.874 | (-0.413 to 2.162) | 0.183 |
| English proficiency → Psychological distress Wave Five | -0.003 | (-0.113 to 0.108) | 0.964 |
| Education → Psychological distress Wave Five |  | |  |
| 6 or fewer years of schooling | Ref | |  |
| 7 to 11 years of schooling | 0.417 | (-0.283 to 1.117) | 0.243 |
| 12 or more years of schooling | -0.158 | (-0.936 to 0.620) | 0.691 |
| Remoteness area → Psychological distress Wave Five |  | |  |
| Major cities | Ref | |  |
| Regional Australia | 0.271 | (-0.650 to 1.193) | 0.564 |
| Migration pathway → Psychological distress Wave Five |  | |  |
| Onshore | Ref | |  |
| Offshore | -0.868 | (-1.694 to -0.042) | 0.039 |
| Main source of income → Psychological distress Wave Five |  | |  |
| Own or spouse/parent's salary, savings | Ref | |  |
| Government payments | 0.933 | (-0.017 to 1.882) | 0.054 |
| Mode of interview → Psychological distress Wave Five |  | |  |
| Computer-assisted self-interview | Ref | |  |
| Computer-assisted personal interview with interviewer | -0.062 | (-0.644 to 0.519) | 0.833 |
| Computer-assisted personal interview with interpreter | -0.662 | (-2.537 to 1.213) | 0.489 |
| Emotional/instrumental support Wave One ↔ Informational support Wave One | 0.016 | (-0.019 to 0.052) | 0.357 |
| Emotional/instrumental support Wave One ↔ Psychological distress Wave One | 0.137 | (-0.096 to 0.369) | 0.249 |
| Informational support Wave One ↔ Psychological distress Wave One | -0.711 | (-0.914 to -0.507) | 0.000 |
| Emotional/instrumental support Wave Three ↔ Informational support Wave Three | 0.027 | (-0.088 to 0.062) | 0.141 |
| Emotional/instrumental support Wave Three ↔ Psychological distress Wave Three | 0.048 | (-0.185 to 0.283) | 0.684 |
| Informational support Wave Three ↔ Psychological distress Wave Three | -0.677 | (-0.874 to -0.479) | 0.000 |
| Emotional/instrumental support Wave Five ↔ Informational support Wave Five | 0.004 | (-0.029 to 0.037) | 0.832 |
| Emotional/instrumental support Wave Five ↔ Psychological distress Wave Five | -0.003 | (-0.240 to 0.234) | 0.979 |
| Informational support Wave Five ↔ Psychological distress Wave Five | -1.208 | (-1.399 to -1.017) | 0.000 |

ꝉ Statistical significance set at *p* < 0.05

**Table 2. Effect of gender on social support and psychological distress (full path model)**

|  | Men, n=1249 | | | Women, n=1015 | | |
| --- | --- | --- | --- | --- | --- | --- |
| Pathway | **Path coef.** | **95% CI** | ***p*-value^ꝉ^** | **Path coef.** | **95% CI** | ***p*-value^ꝉ^** |
| Emotional/instrumental support Wave One → Psychological distress Wave Three | -0.341 | (-0.691 to 0.086) | 0.056 | -0.311 | (-0.715 to 0.092) | 0.130 |
| Informational support Wave One → Psychological distress Wave Three | -0.100 | (-0.484 to 0.284) | 0.610 | 0.128 | (-0.376 to 0.633) | 0.619 |
| Psychological distress Wave One → Psychological distress Wave Three | 0.333 | (0.274 to 0.393) | 0.000 | 0.358 | (0.294 to 0.422) | 0.000 |
| Age → Psychological distress Wave Three | 0.015 | (-0.016 to 0.046) | 0.349 | 0.035 | (0.004 to 0.065) | 0.025 |
| Marital status → Psychological distress Wave Three |  | |  |  | |  |
| No | Ref | |  | Ref | |  |
| Yes | 0.228 | (-0.630 to 1.086) | 0.602 | -0.225 | (-0.979 to 0.528) | 0.558 |
| Birth region → Psychological distress Wave Three |  | |  |  | |  |
| Africa | Ref | |  | Ref | |  |
| Middle East | 2.712 | (1.143 to 4.281) | 0.001 | 2.454 | (0.547 to 4.360) | 0.012 |
| South-East Asia | -0.920 | (-3.035 to 1.195) | 0.394 | -2.323 | (-4.628 to -0.018) | 0.048 |
| Southern Asia | -0.885 | (-2.651 to 0.881) | 0.326 | -2.434 | (-4.689 to -0.179) | 0.034 |
| Central Asia | -0.634 | (-2.312 to 1.045) | 0.460 | 1.221 | (-0.814 to 3.256) | 0.240 |
| English proficiency → Psychological distress Wave Three | -0.141 | (-0.279 to -0.004) | 0.044 | -0.026 | (-0.180 to 0.128) | 0.742 |
| Education → Psychological distress Wave Three |  |  |  |  | |  |
| 6 or fewer years of schooling | Ref | |  | Ref | |  |
| 7 to 11 years of schooling | 0.476 | (-0.424 to 1.376) | 0.300 | 0.106 | (-0.930 to 1.142) | 0.841 |
| 12 or more years of schooling | 0.124 | (-0.856 to 1.104) | 0.804 | 0.253 | (-0.887 to 1.393) | 0.664 |
| Remoteness area → Psychological distress Wave Three |  | |  |  | |  |
| Major cities | Ref | |  | Ref | |  |
| Regional Australia | 2.187 | (0.913 to 3.462) | 0.001 | 1.506 | (0.258 to 2.754) | 0.018 |
| Migration pathway → Psychological distress Wave Three |  | |  |  | |  |
| Onshore | Ref | |  | Ref | |  |
| Offshore | -0.612 | (-1.510 to 0.286) | 0.182 | 2.261 | (0.580 to 3.943) | 0.008 |
| Main source of income → Psychological distress Wave Three |  | |  |  | |  |
| Own or spouse/parent's salary, savings | Ref | |  | Ref | |  |
| Government payments | -0.501 | (-1.531 to 0.529) | 0.340 | -0.009 | (-1.789 to 1.770) | 0.992 |
| Mode of interview → Psychological distress Wave Three |  | |  |  | |  |
| Computer-assisted self-interview | Ref | |  | Ref | |  |
| Computer-assisted personal interview with interviewer | 0.439 | (-0.296 to 1.174) | 0.242 | 0.189 | (-0.667 to 1.045) | 0.665 |
| Computer-assisted personal interview with interpreter | -2.039 | (-5.109 to 1.032) | 0.193 | 0.152 | (-2.148 to 2.451) | 0.897 |
| Emotional/instrumental support Wave One → Psychological distress Wave Five | -0.305 | (-0.674 to 0.063) | 0.104 | -0.064 | (-0.476 to 0.348) | 0.760 |
| Informational support Wave One → Psychological distress Wave Five | 0.029 | (-0.391 to 0.450) | 0.891 | -0.745 | (-1.261 to -0.229) | 0.005 |
| Emotional/instrumental support Wave Three → Psychological distress Wave Five | -0.026 | (-0.426 to 0.374) | 0.898 | 0.035 | (-0.382 to 0.452) | 0.868 |
| Informational support Wave Three → Psychological distress Wave Five | -0.488 | (-0.953 to -0.022) | 0.040 | -0.191 | (-0.702 to 0.318) | 0.462 |
| Psychological distress Wave One → Psychological distress Wave Five | 0.192 | (0.124 to 0.260) | 0.000 | 0.108 | (0.039 to 0.177) | 0.002 |
| Psychological distress Wave Three → Psychological distress Wave Five | 0.247 | (0.175 to 0.318) | 0.000 | 0.332 | (0.260 to 0.405) | 0.000 |
| Age → Psychological distress Wave Five | 0.013 | (-0.022 to 0.047) | 0.471 | 0.032 | (-0.001 to 0.065) | 0.059 |
| Marital status → Psychological distress Wave Five |  | |  |  | |  |
| No | Ref | |  | Ref | |  |
| Yes | -0.075 | (-0.982 to 0.832) | 0.870 | -0.588 | (-1.361 to 0.185) | 0.136 |
| Birth region → Psychological distress Wave Five |  | |  |  | |  |
| Africa | Ref | |  | Ref | |  |
| Middle East | 2.515 | (0.918 to 4.112) | 0.002 | 0.559 | (-1.264 to 2.383) | 0.548 |
| South-East Asia | -0.940 | (-3.174 to 1.294) | 0.409 | -1.444 | (-3.782 to 0.893) | 0.226 |
| Southern Asia | 0.702 | (-1.170 to 2.574) | 0.463 | -1.667 | (-4.010 to 0.677) | 0.163 |
| Central Asia | 0.557 | (-1.159 to 2.274) | 0.525 | 1.082 | (-0.895 to 3.058) | 0.283 |
| English proficiency → Psychological distress Wave Five | -0.054 | (-0.204 to 0.096) | 0.480 | 0.055 | (-0.109 to 0.219) | 0.510 |
| Education → Psychological distress Wave Five |  | |  |  | |  |
| 6 or fewer years of schooling | Ref | |  | Ref | |  |
| 7 to 11 years of schooling | 0.542 | (-0.397 to 1.481) | 0.258 | 0.344 | (-0.702 to 1.390) | 0.519 |
| 12 or more years of schooling | -0.295 | (-1.337 to 0.747) | 0.579 | -0.030 | (-1.195 to 1.135) | 0.960 |
| Remoteness area → Psychological distress Wave Five |  | |  |  | |  |
| Major cities | Ref | |  | Ref | |  |
| Regional Australia | 0.039 | (-1.276 to 1.354) | 0.953 | 0.116 | (-1.176 to 1.408) | 0.861 |
| Migration pathway → Psychological distress Wave Five |  | |  |  | |  |
| Onshore | Ref | |  | Ref | |  |
| Offshore | -1.148 | (-2.123 to -0.172) | 0.021 | -0.541 | (-2.211 to 1.129) | 0.525 |
| Main source of income → Psychological distress Wave Five |  | |  |  | |  |
| Own or spouse/parent's salary, savings | Ref | |  | Ref | |  |
| Government payments | 0.779 | (-0.329 to 1.887) | 0.168 | 1.261 | (-0.556 to 3.077) | 0.174 |
| Mode of interview → Psychological distress Wave Five |  | |  |  | |  |
| Computer-assisted self-interview | Ref | |  | Ref | |  |
| Computer-assisted personal interview with interviewer | -0.140 | (-0.906 to 0.625) | 0.719 | -0.085 | (-0.965 to 0.795) | 0.850 |
| Computer-assisted personal interview with interpreter | -1.812 | (-5.113 to 1.489) | 0.282 | -0.352 | (-2.641 to 1.937) | 0.763 |
| Emotional/instrumental support Wave One ↔ Informational support Wave One | 0.035 | (-0.015 to 0.084) | 0.170 | -0.007 | (-0.055 to 0.042) | 0.786 |
| Emotional/instrumental support Wave One ↔ Psychological distress Wave One | 0.161 | (-0.140 to 0.461) | 0.295 | 0.131 | (-0.226 to 0.488) | 0.471 |
| Informational support Wave One ↔ Psychological distress Wave One | -0.807 | (-1.068 to -0.531) | 0.000 | -0.551 | (-0.844 to -0.259) | 0.000 |
| Emotional/instrumental support Wave Three ↔ Informational support Wave Three | 0.008 | (-0.038 to 0.054) | 0.729 | 0.038 | (-0.014 to 0.091) | 0.154 |
| Emotional/instrumental support Wave Three ↔ Psychological distress Wave Three | 0.048 | (-0.246 to 0.341) | 0.751 | 0.074 | (-0.292 to 0.441) | 0.691 |
| Informational support Wave Three ↔ Psychological distress Wave Three | -0.807 | (-1.068 to -0.547) | 0.000 | -0.509 | (-0.800 to -0.219) | 0.001 |
| Emotional/instrumental support Wave Five ↔ Informational support Wave Five | 0.011 | (-0.033 to 0.055) | 0.621 | -0.003 | (-0.052 to 0.045) | 0.894 |
| Emotional/instrumental support Wave Five ↔ Psychological distress Wave Five | -0.111 | (-0.418 to 0.194) | 0.474 | 0.139 | (-0.217 to 0.494) | 0.445 |
| Informational support Wave Five ↔ Psychological distress Wave Five | -1.315 | (-1.575 to -1.055) | 0.000 | -1.027 | (-1.295 to -0.759) | 0.000 |

^ꝉ^ Statistical significance set at *p* < 0.05

**Table 3: Effect of age group on social support and psychological distress (full path model)**

|  | Age 18 to 29 years old, n=818 | | | Age 30 to 44 years old, n=856 | | | Age 45 to 75 years old, n=590 | | |
| --- | --- | --- | --- | --- | --- | --- | --- | --- | --- |
| Pathway | **Path coef.** | **95% CI** | ***p*-value^ꝉ^** | **Path coef.** | **95% CI** | ***p*-value^ꝉ^** | **Path coef.** | **95% CI** | ***p*-value^ꝉ^** |
| Emotional/instrumental support Wave One → Psychological distress Wave Three | -0.533 | (-0.946 to -0.119) | 0.012 | -0.406 | (-0.858 to 0.047) | 0.079 | -0.004 | (-0.525 to 0.517) | 0.988 |
| Informational support Wave One → Psychological distress Wave Three | 0.085 | (-0.371 to 0.541) | 0.714 | -0.152 | (-0.665 to 0.360) | 0.560 | 0.049 | (-0.608 to 0.706) | 0.884 |
| Psychological distress Wave One → Psychological distress Wave Three | 0.292 | (0.218 to 0.365) | 0.000 | 0.343 | (0.269 to 0.417) | 0.000 | 0.376 | (0.297 to 0.455) | 0.000 |
| Gender → Psychological distress Wave Three |  | |  |  | |  |  | |  |
| Men | Ref | |  | Ref | |  | Ref | |  |
| Women | 0.490 | (-0.356 to 1.336) | 0.256 | 0.997 | (0.104 to 1.891) | 0.029 | 0.779 | (-0.320 to 1.878) | 0.165 |
| Marital status → Psychological distress Wave Three |  | |  |  | |  |  | |  |
| No | Ref | |  | Ref | |  | Ref | |  |
| Yes | 0.285 | (-0.581 to 1.150) | 0.519 | -1.085 | (-2.063 to -0.107) | 0.030 | -0.892 | (-2.145 to 0.361) | 0.163 |
| Birth region → Psychological distress Wave Three |  | |  |  | |  |  | |  |
| Africa | Ref | |  | Ref | |  | Ref | |  |
| Middle East | 3.875 | (1.454 to 6.296) | 0.002 | 3.058 | (1.404 to 4.712) | 0.000 | 2.533 | (-0.354 to 5.421) | 0.086 |
| South-East Asia | 0.413 | (-2.377 to 3.203) | 0.772 | -2.061 | (-4.254 to 0.131) | 0.065 | -2.595 | (-6.410 to 1.219) | 0.182 |
| Southern Asia | 0.927 | (-1.619 to 3.473) | 0.475 | -2.146 | (-4.236 to -0.056) | 0.044 | -2.298 | (-5.833 to 1.237) | 0.203 |
| Central Asia | 1.606 | (-0.900 to 4.111) | 0.209 | -0.144 | (-1.911 to 1.624) | 0.873 | 0.744 | (-2.352 to 3.840) | 0.638 |
| English proficiency → Psychological distress Wave Three | -0.065 | (-0.218 to 0.089) | 0.410 | -0.002 | (-0.179 to 0.174) | 0.978 | -0.170 | (-0.383 to 0.043) | 0.118 |
| Education → Psychological distress Wave Three |  | |  |  | |  |  | |  |
| 6 or fewer years of schooling | Ref | |  | Ref | |  | Ref | |  |
| 7 to 11 years of schooling | -0.893 | (-1.942 to 0.156) | 0.095 | -0.040 | (-1.184 to 1.103) | 0.945 | 2.323 | (0.883 to 3.764) | 0.002 |
| 12 or more years of schooling | -0.548 | (-1.724 to 0.628) | 0.361 | -0.546 | (-1.844 to 0.751) | 0.409 | 1.535 | (0.092 to 2.978) | 0.037 |
| Remoteness area → Psychological distress Wave Three |  | |  |  | |  |  | |  |
| Major cities | Ref | |  | Ref | |  | Ref | |  |
| Regional Australia | 2.007 | (0.702 to 3.311) | 0.003 | 1.813 | (0.328 to 3.299) | 0.017 | 3.010 | (0.972 to 5.049) | 0.004 |
| Migration pathway → Psychological distress Wave Three |  | |  |  | |  |  | |  |
| Onshore | Ref | |  | Ref | |  | Ref | |  |
| Offshore | -0.231 | (-1.444 to 0.983) | 0.710 | 0.635 | (-0.575 to 1.846) | 0.304 | 0.500 | (-1.544 to 2.545) | 0.631 |
| Main source of income → Psychological distress Wave Three |  | |  |  | |  |  | |  |
| Own or spouse/parent's salary, savings | Ref | |  | Ref | |  | Ref | |  |
| Government payments | 0.020 | (-1.227 to 1.266) | 0.976 | -0.619 | (-2.214 to 0.976) | 0.447 | -0.924 | (-2.989 to 1.141) | 0.381 |
| Mode of interview → Psychological distress Wave Three |  | |  |  | |  |  | |  |
| Computer-assisted self-interview | Ref |  |  | Ref |  |  | Ref |  |  |
| Computer-assisted personal interview with interviewer | -0.140 | (-1.116 to 0.836) | 0.778 | 0.740 | (-0.168 to 1.648) | 0.110 | 0.062 | (-0.973 to 1.096) | 0.907 |
| Computer-assisted personal interview with interpreter | -1.295 | (-5.241 to 2.650) | 0.520 | 0.672 | (-1.905 to 3.249) | 0.609 | -1.914 | (-5.477 to 1.650) | 0.293 |
| Emotional/instrumental support Wave One → Psychological distress Wave Five | -0.075 | (-0.516 to 0.365) | 0.738 | -0.295 | (-0.754 to 0.165) | 0.208 | -0.016 | (-0.562 to 0.529) | 0.953 |
| Informational support Wave One → Psychological distress Wave Five | -0.142 | (-0.627 to 0.343) | 0.565 | -0.158 | (-0.697 to 0.383) | 0.567 | -0.890 | (-1.608 to -0.172) | 0.015 |
| Emotional/instrumental support Wave Three → Psychological distress Wave Five | -0.253 | (-0.721 to 0.215) | 0.290 | 0.037 | (-0.457 to 0.531) | 0.884 | 0.086 | (-0.452 to 0.624) | 0.754 |
| Informational support Wave Three → Psychological distress Wave Five | -0.054 | (-0.637 to 0.529) | 0.856 | -0.514 | (-1.073 to 0.045) | 0.071 | -0.507 | (-1.157 to 0.143) | 0.126 |
| Psychological distress Wave Three → Psychological distress Wave Five | 0.269 | (0.176 to 0.361) | 0.000 | 0.295 | (0.214 to 0.376) | 0.000 | 0.309 | (0.215 to 0.402) | 0.000 |
| Gender → Psychological distress Wave Five |  | |  |  | |  |  | |  |
| Men | Ref | |  | Ref | |  | Ref | |  |
| Women | -0.034 | (-0.961 to 0.893) | 0.943 | -0.035 | (-0.959 to 0.890) | 0.940 | 0.142 | (-1.010 to 1.294) | 0.809 |
| Marital status → Psychological distress Wave Five |  | |  |  | |  |  | |  |
| No | Ref | |  | Ref | |  | Ref | |  |
| Yes | 0.048 | (-0.846 to 0.942) | 0.916 | -1.660 | (-2.667 to -0.653) | 0.001 | -0.382 | (-1.697 to 0.923) | 0.566 |
| Birth region → Psychological distress Wave Five |  | |  |  | |  |  | |  |
| Africa | Ref | |  | Ref | |  | Ref | |  |
| Middle East | 1.040 | (-1.241 to 3.321) | 0.371 | 1.452 | (-0.245 to 3.150) | 0.094 | 2.025 | (-0.641 to 4.692) | 0.137 |
| South-East Asia | -2.076 | (-4.826 to 0.674) | 0.139 | -0.518 | (-2.800 to 1.765) | 0.657 | -2.554 | (-6.645 to 1.537) | 0.221 |
| Southern Asia | -0.322 | (-2.820 to 2.176) | 0.801 | 0.648 | (-1.556 to 2.852) | 0.565 | -2.988 | (-6.80 to 0.82) | 0.124 |
| Central Asia | -0.047 | (-2.426 to 2.333) | 0.969 | 0.177 | (-1.649 to 2.003) | 0.849 | 2.580 | (-0.377 to 5.536) | 0.087 |
| English proficiency → Psychological distress Wave Five | 0.098 | (-0.074 to 0.269) | 0.266 | -0.033 | (-0.222 to 0.156) | 0.732 | -0.070 | (-0.292 to 0.152) | 0.537 |
| Education → Psychological distress Wave Five |  | |  |  | |  |  | |  |
| 6 or fewer years of schooling | Ref | |  | Ref | |  | Ref | |  |
| 7 to 11 years of schooling | -0.226 | (-1.326 to 0.874) | 0.687 | 0.842 | (-0.309 to 1.994) | 0.152 | 1.161 | (-0.368 to 2.691) | 0.137 |
| 12 or more years of schooling | -1.360 | (-2.591 to -0.130) | 0.030 | 0.154 | (-1.188 to 1.496) | 0.822 | 0.807 | (-0.721 to 2.335) | 0.301 |
| Remoteness area → Psychological distress Wave Five |  | |  |  | |  |  | |  |
| Major cities | Ref | |  | Ref | |  | Ref | |  |
| Regional Australia | -0.813 | (-2.183 to 0.557) | 0.245 | 1.181 | (-0.299 to 2.662) | 0.118 | 1.370 | (-0.813 to 3.554) | 0.219 |
| Migration pathway → Psychological distress Wave Five |  | |  |  | |  |  | |  |
| Onshore | Ref | |  | Ref | |  | Ref | |  |
| Offshore | -0.508 | (-1.861 to 0.845) | 0.462 | -1.157 | (-2.431 to 0.117) | 0.075 | 0.450 | (-1.501 to 2.400) | 0.651 |
| Main source of income → Psychological distress Wave Five |  | |  |  | |  |  | |  |
| Own or spouse/parent's salary, savings | Ref | |  | Ref | |  | Ref | |  |
| Government payments | 0.952 | (-0.368 to 2.271) | 0.158 | 0.318 | (-1.381 to 2.018) | 0.713 | 1.533 | (-0.584 to 3.651) | 0.156 |
| Mode of interview → Psychological distress Wave Five |  | |  |  | |  |  | |  |
| Computer-assisted self-interview | Ref | |  | Ref | |  | Ref | |  |
| Computer-assisted personal interview with interviewer | 0.612 | (-0.397 to 1.621) | 0.235 | -0.227 | (-1.158 to 0.705) | 0.633 | -0.874 | (-1.960 to 0.213) | 0.115 |
| Computer-assisted personal interview with interpreter | -0.215 | (-4.282 to 3.851) | 0.917 | -1.853 | (-4.475 to 0.769) | 0.166 | 2.130 | (-1.586 to 5.846) | 0.261 |
| Emotional/instrumental support Wave One ↔ Informational support Wave One | 0.013 | (-0.498 to 0.0765) | 0.678 | 0.073 | (0.069 to 0.129) | 0.011 | -0.045 | (-0.104 to 0.013) | 0.130 |
| Emotional/instrumental support Wave One ↔ Psychological distress Wave One | 0.121 | (-0.256 to 0.498) | 0.530 | -0.052 | (-0.417 to 0.313) | 0.779 | 0.429 | (-0.032 to 0.889) | 0.068 |
| Informational support Wave One ↔ Psychological distress Wave One | -0.720 | (-1.058 to -0.381) | 0.000 | -0.606 | (-0.929 to -0.282) | 0.000 | -0.865 | (-1.249 to -0.480) | 0.000 |
| Emotional/instrumental support Wave Three ↔ Informational support Wave Three | 0.074 | (0.010 to 0.137) | 0.023 | 0.037 | (-0.017 to 0.092) | 0.177 | -0.018 | (-0.082 to 0.045) | 0.572 |
| Emotional/instrumental support Wave Three ↔ Psychological distress Wave Three | 0.341 | (-0.061 to 0.743) | 0.097 | -0.004 | (-0.364 to 0.356) | 0.981 | -0.188 | (-0.618 to 0.242) | 0.392 |
| Informational support Wave Three ↔ Psychological distress Wave Three | -0.811 | (-1.138 to -0.485) | 0.000 | -0.789 | (-1.112 to -0.466) | 0.000 | -0.401 | (-0.750 to -0.052) | 0.024 |
| Emotional/instrumental support Wave Five ↔ Informational support Wave Five | -0.041 | (-0.096 to 0.014) | 0.147 | 0.007 | (-0.043 to 0.058) | 0.776 | 0.038 | (-0.026 to 0.103) | 0.246 |
| Emotional/instrumental support Wave Five ↔ Psychological distress Wave Five | 0.098 | (-0.300 to 0.496) | 0.630 | -0.096 | (-0.469 to 0.276) | 0.612 | 0.087 | (-0.328 to 0.501) | 0.682 |
| Informational support Wave Five ↔ Psychological distress Wave Five | -0.979 | (-1.258 to -0.700) | 0.000 | -1.395 | (-1.704 to -1.086) | 0.000 | -1.067 | (-1.454 to -0.680) | 0.000 |

^ꝉ^ Statistical significance set at *p* < 0.05

**Table 4. Effect of migration pathway on social support and psychological distress (full path model)**

|  | Onshore, n=377 | | | Offshore, n=1887 | | |
| --- | --- | --- | --- | --- | --- | --- |
| Pathway | **Path coef.** | **95% CI** | ***p*-value^ꝉ^** | **Path coef.** | **95% CI** | ***p*-value^ꝉ^** |
| Emotional/instrumental support Wave One → Psychological distress Wave Three | -0.207 | (-0.843 to 0.428) | 0.522 | -0.329 | (-0.622 to -0.036) | 0.028 |
| Informational support Wave One → Psychological distress Wave Three | -0.298 | (-0.941 to 0.345) | 0.364 | -0.010 | (-0.357 to 0.336) | 0.953 |
| Psychological distress Wave One → Psychological distress Wave Three | 0.311 | (0.199 to 0.423) | 0.000 | 0.348 | (0.300 to 0.395) | 0.000 |
| Age → Psychological distress Wave Three | 0.042 | (-0.024 to 0.107) | 0.210 | 0.026 | (0.004 to 0.047) | 0.023 |
| Gender |  | |  |  | |  |
| Men | Ref | |  | Ref | |  |
| Women | -1.935 | (-3.721 to -0.148) | 0.034 | 1.059 | (0.505 to 1.614) | 0.000 |
| Marital status → Psychological distress Wave Three |  | |  |  | |  |
| No | Ref | |  | Ref | |  |
| Yes | -0.775 | (-2.051 to 0.501) | 0.234 | -0.051 | (-0.639 to 0.537) | 0.864 |
| Birth region → Psychological distress Wave Three |  | |  |  | |  |
| Africa | Ref | |  | Ref | |  |
| Middle East | 2.896 | (0.117 to 5.674) | 0.041 | 2.828 | (1.354 to 4.301) | 0.000 |
| South-East Asia | -0.30 |  |  | -1.618 | (-3.372 to 0.135) | 0.070 |
| Southern Asia | 0.476 | (-2.922 to 3.873) | 0.784 | -1.989 | (-3.691 to -0.286) | 0.022 |
| Central Asia | -1.485 | (-5.438 to 2.468) | 0.462 | 0.646 | (-0.889 to 2.180) | 0.409 |
| English proficiency → Psychological distress Wave Three | -0.066 | (-0.313 to 0.182) | 0.603 | -0.090 | (-0.203 to 0.023) | 0.117 |
| Education → Psychological distress Wave Three |  | |  |  | |  |
| 6 or fewer years of schooling | Ref | |  | Ref | |  |
| 7 to 11 years of schooling | -1.586 | (-3.380 to 0.208) | 0.083 | 0.455 | (-0.280 to 1.190) | 0.225 |
| 12 or more years of schooling | -0.695 | (-3.039 to 1.650) | 0.561 | 0.168 | (-0.635 to 0.971) | 0.682 |
| Remoteness area → Psychological distress Wave Three |  | |  |  | |  |
| Major cities | Ref | |  | Ref | |  |
| Regional Australia | 1.929 | (0.937 to 4.796) | 0.187 | 2.205 | (1.261 to 3.149) | 0.000 |
| Main source of income → Psychological distress Wave Three |  | |  |  | |  |
| Own or spouse/parent's salary, savings | Ref | |  | Ref | |  |
| Government payments | -1.350 | (-2.752 to 0.052) | 0.059 | 0.027 | (-1.124 to 1.178) | 0.963 |
| Mode of interview → Psychological distress Wave Three |  | |  |  | |  |
| Computer-assisted self-interview | Ref | |  | Ref | |  |
| Computer-assisted personal interview with interviewer | 1.039 | (-0.242 to 2.320) | 0.112 | 0.167 | (-0.448 to 0.782) | 0.595 |
| Computer-assisted personal interview with interpreter | 3.624 |  |  | -0.890 | (-2.810 to 1.030) | 0.364 |
| Emotional/instrumental support Wave One → Psychological distress Wave Five | -0.260 | (-1.491 to 0.971) | 0.679 | -0.174 | (-0.472 to 0.124) | 0.253 |
| Informational support Wave One → Psychological distress Wave Five | 0.431 | (-1.124 to 1.985) | 0.587 | -0.479 | (-0.839 to -0.120) | 0.009 |
| Emotional/instrumental support Wave Three → Psychological distress Wave Five | -0.474 | (-2.830 to 1.882) | 0.693 | 0.037 | (-0.269 to 0.343) | 0.813 |
| Informational support Wave Three → Psychological distress Wave Five | -1.363 | (-2.928 to -0.201) | 0.088 | -0.258 | (-0.625 to 0.110) | 0.169 |
| Psychological distress Wave One → Psychological distress Wave Five | 0.152 | (0.001 to 0.304) | 0.049 | 0.149 | (0.097 to 0.201) | 0.000 |
| Psychological distress Wave Three → Psychological distress Wave Five | 0.202 | (0.011 to 0.394) | 0.038 | 0.302 | (0.248 to 0.356) | 0.000 |
| Age → Psychological distress Wave Five | -0.039 | (-0.094 to 0.015) | 0.158 | 0.033 | (0.009 to 0.056) | 0.007 |
| Gender |  | |  |  | |  |
| Men | Ref | |  | Ref | |  |
| Women | -0.205 | (-5.615 to 5.206) | 0.941 | 0.061 | (-0.519 to 0.642) | 0.836 |
| Marital status → Psychological distress Wave Five |  | |  |  | |  |
| No | Ref | |  | Ref | |  |
| Yes | -0.02 |  |  | -0.670 | (-1.271 to -0.068) | 0.029 |
| Birth region → Psychological distress Wave Five |  | |  |  | |  |
| Africa | Ref | |  | Ref | |  |
| Middle East | 2.969 | (-0.034 to 5.971) | 0.053 | 0.841 | (-0.575 to 2.256) | 0.244 |
| South-East Asia | -6.313 |  |  | -1.695 | (-3.438 to 0.048) | 0.057 |
| Southern Asia | 2.669 |  |  | -1.304 | (-3.010 to 0.401) | 0.134 |
| Central Asia | 1.306 | (-4.510 to 7.122) | 0.660 | 0.304 | (-1.185 to 1.789) | 0.691 |
| English proficiency → Psychological distress Wave Five | -0.125 | (-0.868 to 0.618) | 0.742 | 0.006 | (-0.113 to 0.125) | 0.921 |
| Education → Psychological distress Wave Five |  | |  |  | |  |
| 6 or fewer years of schooling | Ref | |  | Ref | |  |
| 7 to 11 years of schooling | 1.024 | (-0.946 to 2.995) | 0.308 | 0.260 | (-0.487 to 1.007) | 0.496 |
| 12 or more years of schooling | 0.880 | (-3.046 to 4.807) | 0.660 | -0.297 | (-1.127 to 0.534) | 0.484 |
| Remoteness area → Psychological distress Wave Five |  | |  |  | |  |
| Major cities | Ref | |  | Ref | |  |
| Regional Australia | 1.795 | (-2.097 to 5.686) | 0.366 | 0.174 | (-0.802 to 1.150) | 0.727 |
| Main source of income → Psychological distress Wave Five |  | |  |  | |  |
| Own or spouse/parent's salary, savings | Ref | |  | Ref | |  |
| Government payments | 0.199 | (-1.488 to 1.885) | 0.818 | 1.297 | (0.137 to 2.457) | 0.028 |
| Mode of interview → Psychological distress Wave Five |  | |  |  | |  |
| Computer-assisted self-interview | Ref | |  | Ref | |  |
| Computer-assisted personal interview with interviewer | -0.007 | (-2.902 to 2.887) | 0.996 | -0.142 | (-0.776 to 0.491) | 0.660 |
| Computer-assisted personal interview with interpreter | -3.331 |  |  | -0.810 | (-2.757 to 1.136) | 0.415 |
| Emotional/instrumental support Wave One ↔ Informational support Wave One | 0.076 | (-0.016 to 0.167) | 0.106 | 0.010 | (-0.027 to 0.047) | 0.584 |
| Emotional/instrumental support Wave One ↔ Psychological distress Wave One | -0.416 | (-0.913 to 0.082) | 0.102 | 0.224 | (-0.033 to 0.480) | 0.087 |
| Informational support Wave One ↔ Psychological distress Wave One | -0.904 | (-1.371 to -0.437) | 0.000 | -0.642 | (-0.864 to -0.420) | 0.000 |
| Emotional/instrumental support Wave Three ↔ Informational support Wave Three | 0.065 | (-0.011 to 0.140) | 0.093 | 0.015 | (-0.024 to 0.053) | 0.464 |
| Emotional/instrumental support Wave Three ↔ Psychological distress Wave Three | -0.059 | (-0.499 to 0.381) | 0.793 | 0.057 | (-0.202 to 0.317) | 0.666 |
| Informational support Wave Three ↔ Psychological distress Wave Three | -0.700 | (-1.126 to -0.273) | 0.001 | -0.686 | (-0.902 to -0.470) | 0.000 |
| Emotional/instrumental support Wave Five ↔ Informational support Wave Five | 0.049 | (-0.016 to 0.113) | 0.137 | 0.001 | (-0.037 to 0.035) | 0.966 |
| Emotional/instrumental support Wave Five ↔ Psychological distress Wave Five | -0.498 | (-1.004 to 0.089) | 0.054 | 0.032 | (-0.224 to 0.288) | 0.805 |
| Informational support Wave Five ↔ Psychological distress Wave Five | -0.998 | (-1.416 to -0.580) | 0.000 | -1.198 | (-1.404 to -0.992) | 0.000 |

^ꝉ^ Statistical significance set at *p* < 0.05
